# Effect of renal replacement therapy on selected arachidonic acid derivatives concentration

**DOI:** 10.1186/s12882-020-02053-8

**Published:** 2020-09-11

**Authors:** Elżbieta Cecerska-Heryć, Rafał Heryć, Magda Wiśniewska, Natalia Serwin, Bartłomiej Grygorcewicz, Barbara Dołęgowska

**Affiliations:** 1 Department of Laboratory Medicine, Pomeranian Medical University in Szczecin, Powstanców Wielkopolskich 72, 70-111 Szczecin, Poland; 2 Department of Nephrology, Transplantology and Internal Medicine, Pomeranian Medical University in Szczecin, Powstancow Wielkopolskich 72, 70-111 Szczecin, Poland

**Keywords:** Thromboxane B_2_, 5-HETE, 12-HETE, 15-HETE, Renal replacement therapy, Chronic kidney disease, Kidney transplantation

## Abstract

**Background:**

Platelet activation is an important side effect of dialysis, resulted in a subsequent release of arachidonic acid (AA) from activated platelets. AA is involved in many pathologic conditions, such as inflammation, asthma, cancer, diabetes, hypertension, and the pathogenesis of kidney disease. The aim of this study was to define whether the dialysis type affects the concentration of AA derivatives in patients with chronic kidney disease.

**Methods:**

117 patients were qualified to the study group. Based on the type of renal replacement therapy, patients were divided into the following groups: hemodialysis (HD A – before/HD B - after hemodialysis), peritoneal dialysis (PD), kidney transplant patients (TE - before/TE A – after transplantation) and conservative treatment (CT) (30; 30; 27; 30 patients, respectively). The control group consisted of 30 healthy volunteers (NK). The ELISA methods were used to measure the concentrations of TXB2, 5-HETE, 12-HETE, and 15-HETE in the blood serum.

**Results:**

Renal replacement therapy significantly influences the concentration of TXB_2_ (mean ± SD [ng/mL]: HD A- 34.6 ± 9; HD B- 28.3 ± 15.2; PD- 28.3 ± 15.2; CT- 34.2 ± 8.0; TE- 36.7 ± 42.9; TE A- 27.9 ± 8.8; NK– 19.6 ± 15; *p* = 0.010), 5-HETE (mean ± SD [ng/mL]: HD A- 284.2 ± 428.4; HD B- 304.8 ± 516.2; PD – 530.0 ± 553.3; CT- 318.7 ± 366.0; TE- 525.6 ± 358.0; TE A – 409.8 ± 377.1; NK 838.1 ± 497.8; *p* < 0.001) and 15-HETE (HD A—18.1 ± 8.7; HD B- 42.2 ± 14; PD – 36.3 ± 13.8; CT- 33.7 ± 14.0; TE- 19.5 ± 10.2; TE A – 34.4 ± 16.3; NK 22.2 ± 17.8; *p* < 0,001). There was a significant relationship between the type of renal replacement therapy and the duration of dialysis, and the concentration of TXB_2_, 12-HETE acid, and 15-HETE.

**Conclusions:**

The type of renal replacement therapy significantly affects the concentration of AA derivatives. Peritoneal dialysis is the best method of dialysis, taking into account the concentration of arachidonic acid derivatives.

## Background

Chronic kidney disease (CKD) is one of the most frequently occurring diseases that affects 6–15% of the world’s population [1, 2]. Progression of renal failure aggravates ongoing inflammation and increases oxidative stress [3]. Chronic inflammation leads to activation of the endothelium, increased synthesis of adhesion molecules, penetration of monocytes into the intima of the vessels, as well as stimulation of thrombotic processes [4].

Conservative treatment is used in the initial stages of CKD, and dialysis or renal transplantation is necessary at the end-stage. One of the side effects of dialysis is the synthesis of pro-inflammatory factors and increased oxidative stress as a result of the blood contact with artificial materials of dialyzers and the creation of vascular access, which is necessary for dialysis. Consequences of this include transient leukopenia, activation of platelets and cells of the immune and complement systems, and an increase in interleukin-1 concentration [5].

Platelets also play a crucial role during organ transplantation. Many cell-derived blood-borne factors regulate their activation, and to a large extent, depend on the pro-oxidative-antioxidant balance. Healthy endothelium synthesizes prostacyclin (PGI_2_) and nitric oxide to prevent adhesion and activation of platelets. During ischemia and reperfusion of organs undergoing transplantation, there is excessive adhesion of platelets and leukocytes, leading to inflammation and tissue damage. As a result, it can strongly stimulate the immune response, as well as cause an increase in organ alloreactivity [6].

Arachidonic acid (AA) derivatives released from activated platelets are involved in many physical processes. Notably, they take part in the development of inflammation, asthma, cancer, diabetes, hypertension, and the pathogenesis of kidney diseases.

Activated platelets mainly synthesize thromboxane A2 (TXA2) in response to platelet aggregation and vasoconstriction. In solutions, unstable TXA_2_ rapidly degraded to an inactive but more stable form TXB2 [7–9]. Although TXA_2_ appears to be of minor importance in maintaining renal function under physiological conditions, increased TXA_2_ biosynthesis in the kidney was observed in various animal models of kidney disease. Unfortunately, one of the inherent and most important aspects of organ transplantation that occur in the transplanted organ is ischemia-reperfusion injury (I/R) [10]. Several authors have already reported that during I/R injury and allograft rejection, there is increased production of thromboxane synthase and consequently increased thromboxane B_2_ (TXB_2_) concentration. Inhibition of TXA_2_ synthesis during reperfusion significantly improves graft function in animal models of kidney transplantation [11, 12].

The 5-, 12-, and 15- hydroxyeicosatetraenoic (HETE) acids are formed from arachidonic acid by the lipoxygenase pathway [13]. 12-HETE activity is found in platelets as a result of platelet activation by agonists such as thrombin or collagen. The role of the 12-HETE isoform in platelets is not entirely clear, and the role in the direct regulation of platelet function is undocumented. The relationship between HETE acids, chronic kidney disease, platelet activation, and the type of renal replacement therapy used is not yet fully understood. Studies show that lipoxygenases are involved in kidney damage in the course of diabetic nephropathy, and it has been demonstrated that the urine concentration of 12-HETE significantly increases in this group of patients [14, 15]. Besides, 12-HETE, together with 15-HETE, induces the synthesis of TGF-β1 (transforming growth factor β1) in mesangial cells, where its action stimulates the synthesis of extracellular matrix proteins that lead to kidney fibrosis.

Knowledge of the relationship between the type of renal replacement therapy used and the level of circulating arachidonic acid derivatives can be extremely important. It has been shown that during peritoneal dialysis, increased eicosanoids are synthesized by macrophages and peritoneal mesenchymal cells due to the properties of dialysis fluids, which are generally not biocompatible [16]. Understanding the relationship between the level of arachidonic acid derivatives and the type of renal replacement therapy used may inform us on the chances of patient survival post-kidney transplantation, whether dialysis is still providing effective treatment for a patient, or which type of renal replacement therapy is appropriate for a given patient.

## Methods

### Samples

Blood samples (K_2_EDTA) (7,5 ml) and serum (7,5 ml) were drawn from all study participants. Blood was drawn from hemodialysis patients via their arteriovenous fistula, and peripheral venipuncture was used for all other participants (from hemodialysis patients immediately before and after the dialysis and from transplant patient before transplantation and 5–7 days after surgery. K_2_EDTA samples were centrifuged at 2600 rpm for 10 min at 20 °C to obtain plasma, respectively [17]. Serum samples were centrifuged at 6000 rpm for 10 min at 20 °C to obtain serum, respectively.

### Concentrations of arachidonic acid derivatives

The concentrations of TXB_2_, 5-HETE, 12-HETE, 15-HETE were determined by an ELISA (Quantikine® Colorimetric Sandwich ELISAs, R&D Systems, USA, Quantikine® Colorimetric Sandwich ELISAs, My BioSource, USA; Quantikine® Colorimetric Sandwich ELISAs, Cayman Chemical Company, USA).

### Statistical analysis

The Schapiro Wilk test was used to evaluate data distributions, which in the case of some variables (5-HETE concentrations), showed a non-parametric distribution. The Exact Fisher and Chi-square tests were used to analyze quantitative data. A T-test and ANOVA were used for univariate systems, the differences between associated (paired) and unrelated (unpaired) (parametric distribution). The Kruskal-Wallis ANOVA was used to evaluate differences, as well as the U - Test for unpaired data or the Wilcoxon test for paired data (non-parametric distributions). A linear multiple regression model was used to determine the multifactor evaluation of relationships between the parameters studied. Statistical analysis of the results was carried out using Statistica PL 12 Trial (StatSoft) [17].

## Results

The study involved 147 participants, including 117 patients qualified. Based on the type of renal replacement therapy used, patients were divided into the following groups: before (HD A) and after (HD B) hemodialysis (blood was collected from patients immediately before and after dialysis peritoneal dialysis (PD), patients before (TE) and after (TE A) kidney transplantation (5–7 days after the surgery), and conservative treatment (CT) (CKD stages 2–5) (30, 30, 27, and 30 patients, respectively). Patients recruited into the TE group did not simultaneously belong to the group of hemodialysis or peritoneal dialysis patients. The control group consisted of 30 healthy volunteers [17]. Persons belonging to the control group did not have any chronic diseases. In order to qualify for this group, it was to show the basic blood tests such as blood count, biochemistry including, in particular, the concentration of creatinine, albumin, total protein, cholesterol, triglyceride, and glucose levels. Detailed information regarding the study and control can be found in Tables 1 and 2.

**Table 1:** General characteristics of hemodialyzed patients (HD), peritoneal dialysis (PD) treated conservatively (CT), kidney transplantation (TE) and control group (NK) participating in the study (mean ± OS)

Parameters	HD	PD	CT	TE	NK	p*	p**
Gender	M-18	M-16	M-17	M-14	M-18	NS	NS
[M- male; F – female]	K-12	K-14	K-13	K-13	K-12		
Age [years]	63 ± 16	55 ± 15	66 ± 15	57 ± 11	50 ± 8	< 0,001	0,029
Dialysis duration [months]	25 ± 16	26 ± 22	–	54 ± 34	–	–	0,003
Causes of CKD							
1 – DM	5 (17%)	5 (17%)	4 (13%)	1 (4%)	–	–	NS
2 – HA	15 (50%)	3 (10%)	6 (20%)	0 (0%)	–	–	NS
3 – GID	2 (7%)	9 (30%)	6 (20%)	3 (11%)	–	–	NS
4 – ADPKD	0 (0%)	0 (0%)	4 (13%)	2 (7%)	–	–	NS
5 – other	5 (17%)	10 (33%)	4 (13%)	6 (22%)	–	–	NS
6 - unknown	3 (10%)	3 (10%)	6 (20%)	15 (56%)	–	–	NS

P * - statistical significance for differences between HD, PD and CT groups, TE and NK exact Fisher test for qualitative variables; for quantitative variables - one-way ANOVA and;

P ** - statistical significance for differences between HD, PD and CT groups and TE exact Fisher test for qualitative variables for quantitative variables - one-way ANOVA or; DM - diabetic nephropathy; HA - hypertension; GID- glomerular inflammation kidney; ADPKD - polycystic kidney disease inherited autosomal dominant; NS - no statistically significant differences

**Table 2:** General characteristics of hemodialysis patients (A - before, B - after HD), peritoneal dialysis (PD) treated conservatively (CT) before and after kidney transplantation (TE and TE A) and control group (NK) taking partin the study (mean ± OS)

Parameters	HD A	HD B	PD	CT	TE	TE A	NK	P*	P**
Kt/V	1,28 ± 0,21	–	2,77 ± 1,06	–	–	–	–	–	< 0,001
Concentrationof creatinine [mg/dl]	7,9 ± 2,4	3,5 ± 1,3	4,4 ± 2,2	2,5 ± 1,1	7,4 ± 3,3	3,4 ± 2,9	0,8 ± 0,1	< 0,001	< 0,001
Stage of CKD:									
1	0 (0%)	–	0 (0%)	0 (0%)	0 (0%)	–	29 (97%)	–	–
2	0 (0%)		0 (0%)	3 (10%)	0 (0%)		1 (3%)	NS	NS
3	0 (0%)	–	0 (0%)	10 (33%)	0 (0%)	–	0 (0%)	NS	–
4	0 (0)%)	–	0 (0%)	12 (40%)	0 (0%)	–	0 (0%)	–	–
5	30 (100%)	–	31 (100%)	5 (17%)	27 (100%)	–	0 (0%)	–	NS

P * - statistical significance for differences between HD, PD and CT groups, TE and NK for quantitative variables - Kruskal Wallis ANOVA, ANOVA one-way ANOVA or Student’s t test

P ** - statistical significance for differences between HD, PD and CT and TE groups for Kruskal Wallis’s ANOVA quantitative variables or ANOVA one-way analysis

Kt / V - dialysis index (volume fraction V purified by clearance K at time t)

NS - no statistically significant relationships were found

**Fig. 1:**
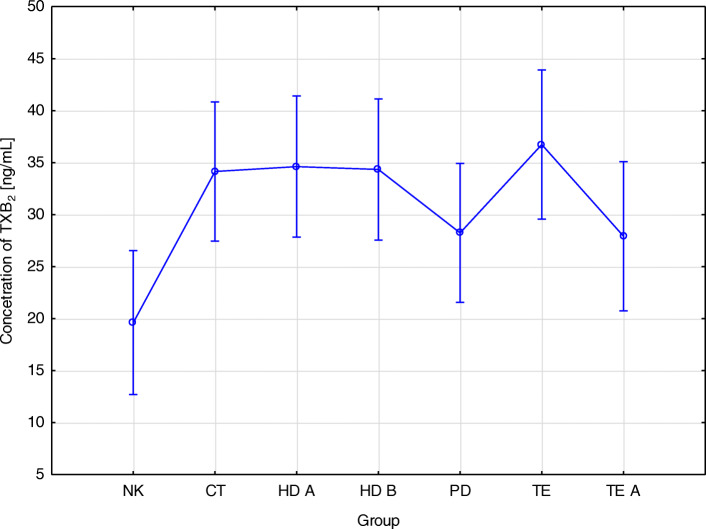
Relationship between the type of renal replacement therapy and the concentration of TXB_2_. TXB_2_ concentration - differences between NK groups, CT, HD A, HD B, PD, TE, TE A (*p* = 0.010). NK- control group CT - treated conservatively; HD A - before hemodialysis; HD B - after hemodialysis; PD - peritoneal dialysis; TE- before kidney transplantation: TE A - after kidney transplantation

Statistically significant relationship between the concentration of TXB_2_ and the studied groups (HD A- 34.6; HD B- 28.3; PD- 28.3; CT- 34.2 ± 8.0; TE- 36.7; TE A- 27.9; NK − 19.6 [ng/mL]; *p* = 0.01) (Table 3) was observed. The lowest TXB_2_ concentration was found in the control group and the highest in patients before kidney transplantation (Fig. 1). Statistically significant differences in the concentration of thromboxane were also demonstrated in the serum of patients before hemodialysis (HD A) and before kidney transplantation (TE), as well as the control group (NK). An association was also found between serum TXB_2_ concentrations in group HD B and TE A and HD B and NK. The relationship between the concentration of thromboxane in the serum of patients treated conservatively, before kidney transplantation, and in the control group, as well as between the peritoneal dialysis and control groups, was also demonstrated. Statistically significant differences were also observed between the concentration of TXB_2_ in the TE and NK groups, and TE A and NK (Table 4).

**Table 3:** Concentration of platelet-derived growth factors of patients with chronic renal disease hemodialysed (before and after HD A, HD B), peritoneal dialysis (PD), conservative treatment (CT), before and after kidney transplantation (TE, TE A) and in the control group (NK) (mean ± OS, median – lower and upper quartile)

Concentration of arachidonic acid derivatives Groups	TXB_2_ [ng/mL]	5-HETE [ng/mL]	12-HETE [ng/mL]	15-HETE [ng/mL]
HD A	34,6 ± 9	284,2 ± 428,4	3,0 ± 0,4	18,1 ± 8,7
	36,9 (29,8; 41,8)	76,8 (1,34; 1465)	2,83 (2,5; 4)	17,0 (4,4; 40,1)
HD B	34,3 ± 10,5	304,8 ± 516,2	3,1 ± 0,6	42,2 ± 14,8
	38,5 (30,7; 40,9)	65,92 (1,11; 1580)	3,0 (1,3; 4,9)	44,9 (18,3; 76,1)
PD	28,3 ± 15,2	530,0 ± 553,3	3,1 ± 2,1	36,3 ± 13,8
	29,8 (17,8; 37,4)	199,4 (11,6; 1511)	3,0 (0,1; 13,2)	38,5 (11,6; 68,6)
CT	34,2 ± 8,0	318,7 ± 366,0	3,0 ± 0,8	33,7 ± 14,0
	35,6 (26,9; 42,3)	151,4 (8,4; 1333,3)	3,2 (0,1; 4,1)	28,8 (13,4; 65,9)
TE	36,7 ± 42,9	525,6 ± 358,0	3,7 ± 1,9	19,5 ± 10,2
	23,16 (14,18; 38,7)	622,6 (25,7; 1219,0)	2,8 (1,8; 8,8)	15,8 (1,9; 43,6)
TE A	27,9 ± 8,8	409,8 ± 377,1	2,8 ± 0,8	34,4 ± 16,3
	26,7 (20,9; 34,7)	265,4 (13,6; 1157)	2,7 (0,1; 4,8)	35,4 (7,2; 73,0)
NK	19,6 ± 15	838,1 ± 497,8	3,3 ± 1,0	22,2 ± 17,8
	18,3 (10,0; 25,5)	792,0 (21,9; 1933,8)	3,1 (1,5; 6,9)	19,4 (0,2; 89,2)
P	0,010	< 0,001	NS	< 0,001

**Table 4:** Statistical differences in the concentration of arachidnonic acid derivatives, between the studied groups (*p*-value)

Groups	HD A	HD B	PD	CT	TE	TE A	NK
TXB_2_							
HD A	–	NS	NS	NS	NS	0,006	< 0,001
HD B	NS	–	NS	NS	NS	0,016	< 0,001
PD	NS	NS	–	NS	NS	NS	NS
CT	NS	NS	NS	–	NS	0,007	< 0,001
TE	NS	NS	NS	NS	–	NS	0,048
TE A	0,006	0,016	NS	0,007	NS	–	0,015
NK	< 0,001	< 0,001	NS	< 0,001	0,048	0,015	–
5-HETE							
HD A	–	NS	0,028	NS	0,004	NS	< 0,001
HD B	NS	–	0,008	NS	0,002	0,017	< 0,001
PD	0,028	0,008	–	NS	NS	NS	0,013
CT	NS	NS	NS	–	0,034	NS	< 0,001
TE	0,004	0,002	NS	0,034	–	NS	NS
TE A	NS	0,017	NS	NS	NS	–	NS
NK	< 0,001	< 0,001	0,013	< 0,001	NS	NS	–
12-HETE							
HD A	–	NS	NS	NS	NS	NS	NS
HD B	NS	–	NS	NS	NS	NS	NS
PD	NS	NS	–	NS	NS	NS	NS
CT	NS	NS	NS	–	NS	0,013	NS
TE	NS	NS	NS	NS	–	NS	NS
TE A	NS	NS	NS	0,013	NS	–	0,048
NK	NS	NS	NS	NS	NS	0,048	–
15-HETE							
HD A	–	< 0,001	< 0,001	< 0,001	NS	< 0,001	NS
HD B	< 0,001	–	NS	0,028	< 0,001	NS	< 0,001
PD	< 0,001	NS	–	NS	< 0,001	NS	0,001
CT	< 0,001	0,028	NS	–	NS	0,013	NS
TE	NS	< 0,001	< 0,001	NS	–	< 0,001	NS
TE A	< 0,001	NS	NS	0,013	< 0,001	–	0,010
NK	NS	< 0,001	0,001	NS	NS	0,010	–

*NS* No significant

**Fig. 2:**
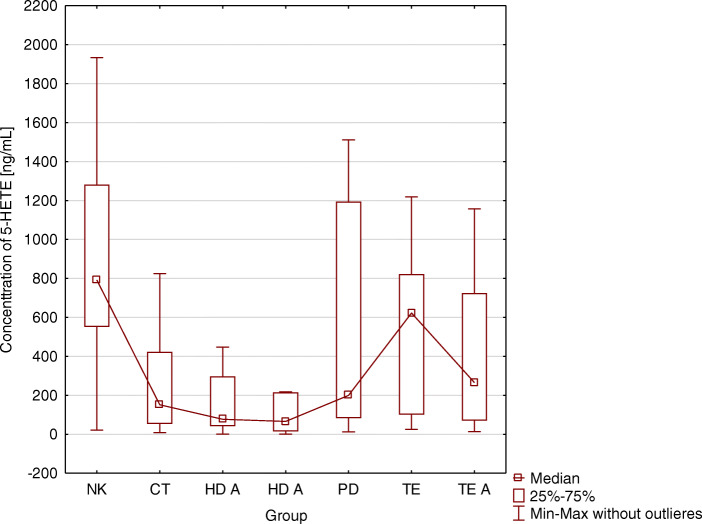
Relationship between the type renal replacement therapy and the concentration of 5-HETE. 5-HETE concentration - differences between CT, HD A, HD B, PD, TE, TE A, NK groups (*p* < 0,001). NK - control group CT - treated conservatively; HD A - before hemodialysis; HD B - after hemodialysis; PD - peritoneal dialysis; TE - before kidney transplantation: TE A - after kidney transplantation

There were statistically significant differences between the concentrations of 5-HETE acid in the blood serum of patients in different study groups (*p* < 0.001; (HD A- 284.2; HD B- 304.8;PD - 530.0; CT- 318.7; TE- 525.6; TE A - 409.8; NK- 838.1 [ng/mL]; *p* < 0.001) (Table 3). The lowest concentration of 5-HETE was obtained in patients before hemodialysis, and the highest was in the control group (Fig. 2). Differences in the concentration of 5-HETE acid between HD A and PD groups, and TE and NK groups were observed, as well as between HD B and PD groups, TE and TE A groups, and between HD B and NK groups. There was also a relationship between the concentration of 5-HETE acid in the blood serum of patients with peritoneal dialysis and control group, and before and after kidney transplantation with the control group, as well as between patients treated conservatively (CT) and before kidney transplantation (TE) and the control group (NK) (Table 4).

There was no relationship between serum 12-HETE concentrations between the study groups (HD A- 3.0; HD B- 3.1; PD- 3.1; CT- 3.0; TE- 3.7; TE A- 2.8; NK 3.3 [ng/mL]; *p* > 0.05) (Table 3). Statistically significant differences occurred between the concentration of 12-HETE in blood serum of patients treated conservatively (CT) and after renal transplantation (TE A) (Table 4), and between the group of patients after transplantation and control group (Table 4).

**Fig. 3:**
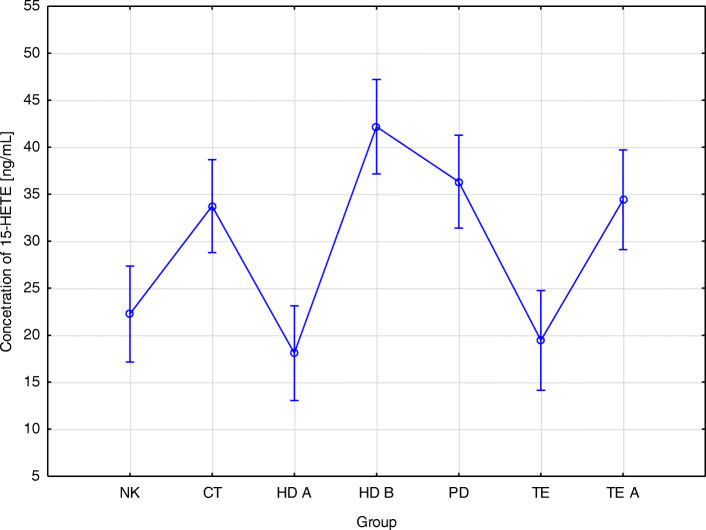
Relationship between the type of renal replacement therapy and the concentration of 15-HETE. 15-HETE concentration - differences between NK groups, CT, HD A, HD B, PD, TE, TE A (*p* < 0,001). NK- control group CT - treated conservatively; HD A - before hemodialysis; HD B - after hemodialysis; PD - peritoneal dialysis; TE- before kidney transplantation: TE A - after kidney transplantation

We found a statistically significant differences between the concentrations of 15-HETE acid in the blood serum of patients in different study groups (HD A- 18.1; HD B- 42.2; PD- 36.3; CT- 33.7; TE- 19.5; TE A- 34.4; NK- 22.2 [ng/mL]; *p* < 0.001) (Table 3). The lowest concentrations of 15-HETE acid were observed in patients before hemodialysis and in the control group, and the highest concentration was in the group of patients after hemodialysis (Fig. 3). There were also statistically significant differences between the concentration of 15-HETE in the serum of patients before hemodialysis (HD A) and peritoneal dialysis (PD), treated conservatively, and after renal transplantation (TE A). The analysis shows a relationship between 15-HETE acid concentration in HD B and CT, HD B and TE, and HD B and NK groups. Significant differences in the concentration of 15-HETE acid occurred in patients on peritoneal dialysis, before kidney transplantation, and in the control group, as well as between transplant patients and the control group. There were significant differences in the concentration of 15-HETE acid in the blood serum of patients before and after hemodialysis, and before and after renal transplantation (Table 4).

The concentration of arachidonic acid derivatives is influenced by sex, duration of dialysis, and the cause and severity of CKD.

We found a correlation between TXB_2_, 12-HETE acid, and 15-HETE acid concentrations, the type of renal replacement therapy used, and the duration of dialysis (*p* < 0.001). There were also statistically significant differences between the concentration of the examined parameters, the type of renal replacement therapy, and the age of the patient (*p* < 0.001) (Table 5). We also observed a relationship between the concentration of 5-HETE acid in the blood serum of patients and the stage of chronic kidney disease (*p* = 0.003) (Table 5). 5-HETE concentration was highest in patients in the second stage of CKD, and lowest in patients in the fifth stage.

**Table 5:** The influence of particular parameters on concentrations of arachidonic acid derivatives

Parameters	TXB_2_	5-HETE	12-HETE	15-HETE
Gender	NS	NS	NS	NS
Renal replacement therapy and duration of dialysis	< 0,001	NS	< 0,001	< 0,001
Renal replacement therapy and age	< 0,001	< 0,001	< 0,001	< 0,001
Stage of CKD	NS	0,003	NS	NS
Causes of CKD	NS	NS	NS	NS

The table presents *p* values ​​defining statistical significance. The relationship between gender, duration of dialysis, age, stage of chronic kidney disease and the causes of chronic kidney disease and the concentrations of arachidnonic acid derivatives was assessed using one-way ANOVA

HD and duration of dialysis - the relationship between the type of therapy (hemodialysis, peritoneal dialysis, patients before kidney transplantation), duration of dialysis and the concentrations of arachidnonic acid derivatives

HD and age - dependence between the studied groups (hemodialysis, peritoneal dialysis, conservative treatment, patients before kidney transplantation and control group), patients’ age and concentrations of arachidnonic acid derivatives

The stage of chronic kidney disease - the relationship between the severity of chronic kidney disease based on eGFR and the activity of concentrations of arachidnonic acid derivatives

The causes of chronic kidney disease - the relationship between selected causes of chronic disease and the concentrations of arachidnonic acid derivatives

NS - no statistically significant relationship was found

Based on multivariate regression analysis, it was found that parameters such as the type of renal replacement therapy, age of patients, duration of dialysis, causes, and stage of CKD had an effect on thromboxane concentration at approximately 37% (Table 6).

**Table 6:** Analysis of the impact of the tested parameters on the concentration and activity values ​​of the tested parameters - multifactorial regression analysis

Dependent variable	Independent variable	β	*R*^2^	p	p for the model	F
TXB_2_	Renal replacement therapy	−4,65	0,37	0,042	< 0,001	2,88
	Age	−11,75		0,07		
	Duration of dialysis	−64,57		0,004		
	Stage of CKD	0,28		0,056		
	Causes of CKD	−18,36		0,14		

*β* Standardized coefficient in the regression equtation, *R*^*2*^ coefficient of determination, *p p*-value, *TXB*_*2*_ Tromboxan

## Discussion

In patients with chronic kidney disease, platelets are activated as a consequence of dialysis, increased inflammation, and oxidative stress, which results in an increased production of thromboxane and HETE acids [5]. It has also been shown that peritoneal dialysis leads to an increased synthesis of eicosanoids by peritoneal macrophages and mesenchymal cells due to the properties of the dialysis fluid, which are generally not biocompatible [16]. Therefore, knowledge of the relationship between the type of renal replacement therapy and the level of arachidonic acid derivatives is extremely important. According to the literature, it is possible to use this knowledge to gain insights into the chances of survival of a patient following kidney transplantation, whether dialysis is still providing effective treatment, or which type of renal replacement therapy is suitable for a given patient.

Activated platelets mainly synthesize thromboxane A2 in response to platelet aggregation and vasoconstriction. Thromboxane A2 is characterized by very short half-life (about 30 s). In aqueous solutions, TXA_2_ is unstable and rapidly degraded to an inactive but more stable form of TXB_2_. Although TXA_2_ appears to be of minor importance in maintaining renal function under physiological conditions, increased TXA_2_ biosynthesis in the kidney is confirmed in various animal models of kidney disease. One of the most important events that occur in a transplanted organ is ischemia-reperfusion injury (I/R), which, unfortunately is an inherent aspect of transplantation. The mechanism of I/R damage includes activation of the inflammatory response, formation of reactive oxygen species, and microcirculation disorders. In addition, several mediators such as TNF-α, endothelin, and arachidonic acid eicosanoid metabolites contribute to such mechanisms, including hydroxyeicosatetraenoic (HETE) acids and thromboxane [10]. Several authors have already reported that during I/R injury and allograft rejection, there is increased production of thromboxane synthase and consequently increased TXB_2_ concentration. Inhibition of TXA_2_ synthesis during reperfusion significantly improves graft function in animal models of kidney transplantation [10, 12].

TXA_2_ is synthesized by mesangial cells and podocytes. Decrease of renal blood flow to afferent and efferent arterioles caused by glomerulonephritis, cyclosporin overdose, or renal transplant rejection. As a consequence, there is a decrease in the number of mesangial cells, increase in the activity of the plasminogen activator inhibitor-1 (PAI-1), decrease in the activity of the tissue plasminogen activator (t -PA) and increase in the level of TGF-β. This leads to the deposition of fibrin and matrix proteins in the glomeruli and the mesangial matrix, which leads to worsening of renal failure [18, 19].

In our study, a statistically significant relationship was found between the concentration of thromboxane B_2_ and the groups studied. The lowest concentration of TXB_2_ was found in the control group and the highest in patients before renal transplantation. Statistically significant differences were also observed between the concentration of TXB_2_ in the TE and NK groups and TE A and NK. In patients after kidney transplantation, a not statistically significant decrease in thromboxane concentration was observed. There was no statistically significant difference between TXB_2_ concentration in patients before or after hemodialysis. On the other hand, there were significant differences between the concentration of thromboxane in hemodialysis patients and peritoneal dialysis, with a lower concentration of TBX_2_ in the PD group.

In the literature, there are many reports on the concentration of thromboxane in patients with different types of renal replacement therapy. Dołęgowska et al. (2009) showed that kidney transplantation is associated with changes in TXB_2_ concentration and that thromboxane alone may be a marker of organ function. In addition, after kidney transplant patients were divided into three groups: early graft function (EGF), slow graft function (SGF), and delayed graft function (DGF), the authors showed that the concentration of thromboxane increased within the first 5 min after transplantation in each of these groups [10]. However, our own studies showed a decrease in TXB_2_ concentration within a few days after kidney transplantation. Averna et al. (2001) showed that the administration of drugs that reduce or eliminate thromboxane-dependent activation of platelets in vivo might reduce the risk of cardiovascular events but may also prevent the long-term survival of patients with kidney transplantation [20]. Considering that an increase in thromboxane concentration may be indicative of transplant rejection and may lead to an increase in TGF-β concentration, which is also a sign of poor functioning of the transplanted kidney [18, 19], together with the results obtained in our own studies, it can be suggested that the decrease in TXB_2_ and TGF-β after kidney transplantation may be a sign of good prognosis for this group of patients. The relationship between the concentration of thromboxane and TGF-β is also confirmed by correlations obtained in our own studies. A positive correlation was found between the concentrations of thromboxane and TGF-β and platelet-derived growth factor-B (PDGF-B) in patients treated conservatively, as well as the positive correlation between PDGF-B and TXB_2_ concentrations after renal transplantation, and a strong positive correlation between the concentration of thromboxane and TGF-β in the control group. Orlińska et al. (1995) showed that both exogenous and endogenous transforming growth factor (TGF) regulates the production of thromboxane, And elevated levels of TGF-β lead to the increased production of TXB_2_ [21].

The data also supports the hypothesis that a lower concentration of TXB_2_ and TGF-β after transplantation is a good prognosis for these patients because no significant correlation was found between TXB_2_ and TGF-β after kidney transplantation. Stępniewska et al. (2019) observed a significantly lower concentration of thromboxane in hemodialysis patients than in peritoneal dialyzes patients and in those treated conservatively (stage 3–5). They also showed that the type of renal replacement therapy affects the concentration of arachidonic acid metabolites, and the concentrations of thromboxane Moreover, they showed that the levels of 20-HETE and 15-HETE acids can be indicators of kidney damage and possible cardiovascular diseases [18]. Zhao et al. (2015), in turn, found that in patients on peritoneal dialysis, there was an increased synthesis of eicosanoids by macrophages and peritoneal mesenchymal cells due to the properties of dialysis fluids, which are generally not biocompatible [16].

In other studies, however, the platelets of patients undergoing regular hemodialysis have been shown to be exposed to increased oxidative stress due to endothelial damage and carbohydrate and lipid metabolism disorders. They are activated excessively because their function is weakened due to ineffective antioxidant activity [22, 23]. Platelets are the main source of TXB_2_, and so the excessive activation of platelets may lead to an increased release of TXB_2_.

In our own studies, thromboxane concentrations were low in the PD group and significantly higher in the groups before and after hemodialysis, as well as in patients treated conservatively. This may support the thesis that platelets are excessively activated during hemodialysis and that these patients are more exposed to oxidative stress than PD patients.

The 5-, 12-, and 15-HETE acids are formed from arachidonic acid on the lipoxygenase pathway [13]. The relationship between HETE acids, chronic kidney disease, and platelet activation and the type of renal replacement therapy is not yet fully understood. Studies show that lipoxygenases are involved in kidney damage in the course of diabetic nephropathy, and the concentration of 12-HETE acid in urine significantly increases in this group of patients. Higher expression of 12/15 LOX (12/15 lipoxygenase) is associated with an increase in fibronectin and other mediators of diabetic nephropathy [14, 15].

According to the latest research, HETE acids can have a strong influence on the intensity of the inflammatory process. For example, the levels of 12-HETE, 15-HETE stimulate the overexpression of pro-inflammatory genes in macrophages, while the levels of 5-HETE promote the production of T cells. Moreover, 12-HETE together with 15-HETE induces the synthesis of TGF-β1 in mesangial cells, where it stimulates the synthesis of proteins of the extracellular matrix, thus leading to renal fibrosis. Due to the very short half-life of HETE acids and its unstable nature, the activities of HETE acids only occur in an autocrine or paracrine manner [24–28].

AA lipoxygenase derivatives are also involved in the regulation of blood pressure. Increased urinary excretion of 12-HETE was found in patients with essential arterial hypertension [11]. HETE acids may also affect early kidney transplantation as evidenced by significant changes in 5-HETE, 12-HETE and 15-HETE levels after kidney transplantation [24, 29]. Matsuyama et al. (2004) reported that the activity of AA derivatives formed on the cyclooxygenase and lipoxygenase pathway correlates with the intensity of I/R [24, 30]. In addition, several other authors report that elevated HETE levels in animals were detected during allograft rejection. These observations clearly justify the need to study this pathway of AA metabolism during human kidney transplantation [16, 24, 31, 32].

Wang et al. [33] have shown that free fatty acids (FFA) are best removed during low flow hemodialysis. As much as 60% of FFA is removed from the plasma after 4 h of hemodialysis. Lipids with a higher molecular weight, such as triglycerides and sphingomyelin, are not effectively removed. The concentration of FFA and SFA (saturated fatty acids) is increased between successive hemodialysis procedures, which is crucial to prevent the risk of cardiovascular events [33]. During peritoneal dialysis, however, there is an increased synthesis of eicosanoids by macrophages and peritoneal mesenchymal cells due to the properties of dialysis fluids, which are generally not biocompatible. The volume and nutritional status of peritoneal dialysis patients also affect the plasma lipid profile and are associated with inflammatory biomarkers (e.g., isoprostanes) and oxidative stress [22]. In our own studies, confirmation was obtained in the form of significantly higher concentrations of 5-HETE and 15-HETE in the group PD than in the group before hemodialysis, and also after hemodialysis in the case of 5-HETE.

Stępniewska et al. (2017) did not demonstrate a relationship between the concentration of 5- and 15-HETE acids and the type of renal replacement therapy used [18]. In the studies described in this work, however, this relationship was found. The lowest concentration of 5-HETE was observed in patients before hemodialysis and the highest in the control group. In the case of 15-HETE acid, the lowest concentration was observed in patients before hemodialysis and in the control group, and the highest concentration in the group of patients after hemodialysis. Reinhold et al. (2013) studied the relationship between concentrations 12- and 15-HETE and the function of a transplanted kidney. In this study, they observed a correlation between the concentration of HETE acids and kidney function 2 weeks post-transplantation but did not find a relationship between 12- and 15-HETE concentrations and acute transplant rejection [34, 35].

Dołęgowska et al. (2010) showed significant perioperative changes in the metabolism of AA derivatives arising in the LOX pathway, expressed as changes in 5-, 12- and 15-HETE concentrations, accompany kidney transplants in humans. These changes relate to early renal function after transplantation (EGF). Moreover, concentrations of 5-, 12- and 15-HETE decreased in the first 5 min after transplantation in the EGF and DGF groups with the exception of the SGF group [24]. HETE acids may, in the future, serve as a new perioperative predictor of early organ function after transplantation. Dołęgowska et al. (2010), however, confirmed the results obtained by Reinhard et al. (2013) showing no relationship between HETE concentration and acute rejection of the transplant. This study also updates the hypothesis previously suggested by other scientists, which is that knowledge of AA metabolism in the early phase of allograft reperfusion may offer an entirely new way to attenuate reperfusion injury during organ transplantation in humans [16].

In our study, there was a significant relationship between 5-HETE concentration before and after kidney transplantation and the control group. The concentration of 5-HETE was highest in the control group and lowest after kidney transplantation. There was also a significant difference between 15-HETE concentration before and after kidney transplantation, with an increase in the concentration of 15-HETE acid after kidney transplantation. There was no relationship between serum 12-HETE concentrations between the groups. However, a significantly lower concentration of 12-HETE acid after renal transplantation was demonstrated compared to the control group and patients treated conservatively. Considering the results obtained by other scientists, and taking into account the high importance of these acids in the body’s inflammatory response, an increase in the concentration of HETE acids after transplantation may indicate poor functioning of a transplanted kidney.

Based on our results, it is difficult to state clearly whether the concentration of HETE acids can indicate the possibility of graft rejection or not, due to the increase in 15-HETE acid concentration and decrease in 5-HETE acid concentration after kidney transplantation, and the lower concentration of 12-HETE compared, for example, compared with patients treated conservatively or the control group. Long-term observation of patients after kidney transplantation would be necessary to fully understand the importance of HETE acids in predicting graft rejection.

It should also be pointed out that eicosanoids are mediators of inflammation, and can also influence the metabolism of fibroblasts in wound healing processes and the reorganization of connective tissue [35]. The creation of vascular access in hemodialysis patients would also explain the increase in systemic compounds whose function is the chemotaxis of fibroblasts and the stimulation of healing processes.

The present study describes the relationship between the thromboxane, 12-HETE, and 15-HETE concentration and the duration of dialysis and the type of therapy used. It has been shown that the longer the dialysis takes, the lower concentration of TXB_2_ and 15-HETE acid. In the case of 12-HETE acid, its highest concentration occurred in patients before kidney transplantation, who, on average, had the most prolonged duration of dialysis. Also, based on a multivariate regression analysis, it was found that parameters such as the type of renal replacement therapy, age of patients, duration of dialysis, cause, and stage of CKD affected thromboxane concentration in 37%. The type of renal replacement therapy (*p* = 0.042) and dialysis duration (*p* = 0.004) both had a significant effect on the concentration of thromboxane.

The results obtained in this study do not confirm those obtained by other scientists, except the concentration of 12-HETE, which indicates increased activation of platelets in patients undergoing long-term dialysis. Because increased production of thromboxane leads to the deposition of fibrin and matrix proteins in the glomeruli and mesangium, and subsequently leads to the worsening of renal failure [18, 19], a lower thromboxane concentration is an indicator of good prognosis for patients on long-term dialysis. A decreasing concentration of 15-HETE might indicate a lower chance of graft rejection after transplantation.

There are no reports on the dependence of 5-HETE on the subject of chronic disease and the stage of chronic kidney disease. It has been shown, however, that supplementation with polyunsaturated fatty acids (PUFA) over 8 weeks results in a decrease in the pro-inflammatory leukotriene-B4 (LTB4) and 5-HETE, and increases the synthesis of less inflammatory leukotriene, LTB5 and 5-hydroperoxyeicosatetraenoic (5-HPETE) in patients with CKD stages 2–5 [36]. In our research, the concentration of 5-HETE decreased as kidney disease progressed. This may indicate less pro-inflammatory processes and a good prognosis for patients in the fifth stage of CKD.

## Conclusions

The type of renal replacement therapy used significantly affects the level of arachidonic acid derivatives (TXB2, 5-HETE, and 15-HETE). Apart from the type of therapy used in CKD patients, the factors significantly affecting the release of arachidonic acid derivatives were the age of patients, the duration of dialysis, the cause of CKD, and the stage of its advancement. Considering the concentration of arachidonic acid derivatives, peritoneal dialysis is the best method of dialysis. Concentrations of arachidonic acid derivatives in patients with CKD may be helpful in the selection of appropriate renal replacement therapy, prognosis of patients after kidney transplantation or long-term dialysis.

## Data Availability

The datasets generated and/or analysed during the current study are not publicly available due [these are sensitive data owned by the Pomeranian Medical University in Szczecin] but are available from the corresponding author on reasonable request.

## Abbreviations

12/15 LOX: 12/15 lipoxygenase
12-HETE: 12- hydroxyeicosatetraenoic acid
15-HETE: 15- hydroxyeicosatetraenoic acid
5-HETE: 5-hydroxyeicosatetraenoic acid
AA: Arachidonic acid
CKD: Chronic kidney disease
CT: Conservative treatment
DGF: Delayed graft function
EGF: Early graft function
FFA: Fatty acids
HD A: Before hemodialysis group
HD B: After hemodialysis group
I/R: Ischemia-reperfusion injury
LOX: Lipoxygenase
LTB4: Pro-inflammatory leukotriene-B4
NK: Control group
PAI-1: Plasminogen activator inhibitor-1
PD: Peritoneal dialysis
PGI_2_: Prostacyclin I_2_
PUFA: Polyunsaturated fatty acids
SGF: Slow graft function
TE: Before transplantation group
TE A: After transplantation group
TGF-β1: Transforming growth factor β1
t-PA: Tissue plasminogen activator
TXA_2_: Thromboxane A_2_
TXB_2_: Thromboxane B_2_
